# Understanding and overcoming the pitfalls and biases of next-generation sequencing (NGS) methods for use in the routine clinical microbiological diagnostic laboratory

**DOI:** 10.1007/s10096-019-03520-3

**Published:** 2019-03-05

**Authors:** Stefan A. Boers, Ruud Jansen, John P. Hays

**Affiliations:** 1 Department of Medical Microbiology and Infectious Diseases, Erasmus University Medical Centre Rotterdam (Erasmus MC), Rotterdam, The Netherlands; 2 Department of Molecular Biology, Regional Laboratory of Public Health Kennemerland, Haarlem, The Netherlands

**Keywords:** Routine clinical microbiological diagnostics, Microbiota analysis, Pitfalls and biases, 16S rRNA gene, (Shotgun) metagenomics, Next-generation sequencing

## Abstract

Recent advancements in next-generation sequencing (NGS) have provided the foundation for modern studies into the composition of microbial communities. The use of these NGS methods allows for the detection and identification of (‘difficult-to-culture’) microorganisms using a culture-independent strategy. In the field of routine clinical diagnostics however, the application of NGS is currently limited to microbial strain typing for epidemiological purposes only, even though the implementation of NGS for microbial community analysis may yield clinically important information. This lack of NGS implementation is due to many different factors, including issues relating to NGS method standardization and result reproducibility. In this review article, the authors provide a general introduction to the most widely used NGS methods currently available (i.e., targeted amplicon sequencing and shotgun metagenomics) and the strengths and weaknesses of each method is discussed. The focus of the publication then shifts toward 16S rRNA gene NGS methods, which are currently the most cost-effective and widely used NGS methods for research purposes, and are therefore more likely to be successfully implemented into routine clinical diagnostics in the short term. In this respect, the experimental pitfalls and biases created at each step of the 16S rRNA gene NGS workflow are explained, as well as their potential solutions. Finally, a novel diagnostic microbiota profiling platform (‘MYcrobiota’) is introduced, which was developed by the authors by taking into consideration the pitfalls, biases, and solutions explained in this article. The development of the MYcrobiota, and future NGS methodologies, will help pave the way toward the successful implementation of NGS methodologies into routine clinical diagnostics.

## Background

The detection, identification, and characterization of pathogenic microorganisms is the major step in establishing appropriate (antimicrobial) treatment for infectious diseases. For this, routine clinical microbiological diagnostic laboratories are equipped with a large arsenal of culture-dependent and culture-independent methods to investigate the etiology of microbial infections. However, the causative agent of an infection may not always be detected using culture-dependent methods, as many microorganisms require specific growth conditions that cannot be (easily) mimicked within a laboratory environment [1]. In addition, most culture-independent methods (e.g., PCR) require a priori knowledge of microorganisms that are suspected to be present within a clinical sample under investigation in order to detect them and, therefore, unexpected microorganisms could evade detection using these culture-independent methods [2]. For these reasons, new culture-independent diagnostic tests are needed to improve the etiological diagnosis in infectious diseases, leading to an improvement in clinical outcomes for patients, better antimicrobial stewardship, improved detection and tracking of disease outbreaks, the detection of viable but non-culturable (VBNC) or other difficult-to-culture microorganisms, and investigations of previously unknown pathogens [3].

One culture-independent technology that has been widely utilized in microbiology research, but not in routine clinical microbiological diagnostics, is next-generation sequencing (NGS). The application of NGS technology and its various methodological variants now makes it possible to detect different types of microorganisms present within a microbial sample simultaneously, using a culture-independent approach and in a single sequencing run [4]. Over the last two decades, such NGS methods have been used extensively in research studies, particularly focusing on the human microbiota and its association with (health and) disease, generating hundreds of publications. For example, there is a tremendous amount of (circumstantial) evidence available that suggests a role for the human gut microbiota in such diseases as allergic diseases [5–7], inflammatory bowel diseases [8, 9], metabolic diseases [10, 11], and even mental diseases [12]. To date however, there has been little focus on the development and application of NGS methods for use in the routine clinical microbiological diagnostic laboratory even though several studies have already shown that the employment of such methods may lead to an improved detection of difficult-to-culture bacteria, for example obligate anaerobic bacteria, in clinical samples [13–16]. Further, obligate anaerobes are known to cause serious infections, yet their detection may be sub-optimal within routine clinical microbiological diagnostic laboratories using traditional specimen collection and detection techniques, as special precautions are required to help preserve the anaerobic environment during specimen collection and transport, and the laboratory needs to provide (potentially unknown) growth components for culture-based detection methods [17]. Therefore, the adaptation of collection, transport, and culture-independent NGS methods could play a major role in the detection and identification of anaerobic infections, or indeed any other infection caused by fastidious or viable but non-culturable (VBNC) microorganisms—examples of VBNCs include antibiotic ‘damaged’ microorganisms that may be present within patients during antimicrobial therapy [18]. A second important point is that obtaining a comprehensive overview of the microbiota within clinical samples means that the microbial community per se could be taken into account when making clinical decisions [19]. Taken together, the adoption of NGS methods within the routine clinical microbiological diagnostic laboratory could be advantageous for both clinicians and patients. However, if the promise of NGS methods is to be achieved, then issues relating to methodological standardization, reproducibility, and the quality of the results obtained need to be addressed [20].

In this review article, the authors provide a general introduction to current NGS methodologies available for the culture-independent detection, identification, and characterization of microorganisms, including their potential pitfalls and biases that can influence the quality and interpretation of the results obtained. This knowledge will provide the reader with a reference for further understanding the current barriers and solutions to the implementation of NGS methods into the routine clinical microbiological diagnostic laboratory. In this respect, a recently published, novel NGS microbiota profiling platform (‘MYcrobiota’) is outlined, which was developed by the authors as a promising first step in the transition of NGS methods into routine clinical microbiological diagnostics.

## NGS methodologies for characterizing microbial communities in clinical samples

**Fig. 1:**
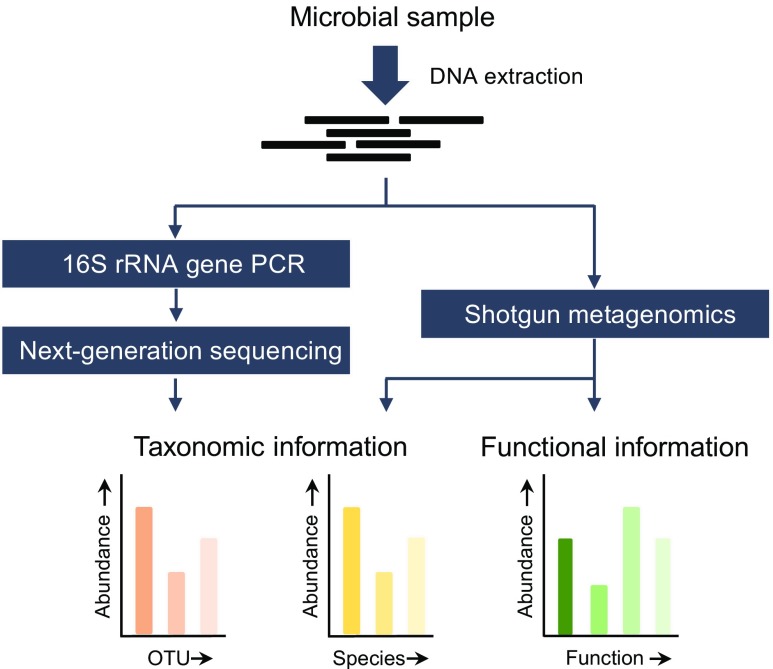
General overview of 16S rRNA gene NGS and shotgun metagenomics methods. Both methods start with the extraction of nucleic acids from a microbial sample. Next, the extracted DNA is either subjected to 16S rRNA gene PCR amplification (16S rRNA gene NGS) or sheared into small DNA fragments (shotgun metagenomics). The resultant 16S rRNA gene amplicons, or sheared DNA fragments, are sequenced using NGS techniques. Finally, all sequence data are processed using an extensive array of bioinformatics algorithms that allows the researcher to explore the taxonomic composition and/or the functional capacity of the sample tested. *OTU* operational taxonomic units—a group of very similar sequences.

The advent of NGS has enabled researchers to investigate the composition and function of microbial populations in very diverse environments with unprecedented resolution and throughput. Currently, the majority of these investigations apply NGS by focusing on either (1) targeted amplicon sequencing, usually using the 16S ribosomal RNA (rRNA) gene as a phylogenetic target (i.e., 16S rRNA gene NGS); or (2) shotgun metagenomics, sequencing the genetic material present within a sample directly using a PCR-independent approach. A general overview of both methods is shown in Fig. 1.

### Targeted amplicon sequencing

Targeted amplicon sequencing is a widely used approach for characterizing microbial communities. Here, DNA is extracted from a (clinical) sample and subjected to PCR amplification using a PCR primer set that targets a taxonomically informative gene that is common to either the prokaryotes (bacteria and archaea), or common to the microbial eukaryotes (fungi or protists)—there is no universal target gene present in both prokaryotic and eukaryotic kingdoms. After amplification, the resultant amplicons are sequenced and then characterized using bioinformatics tools, which search reference sequence databases to determine which microorganisms are present in the sample and at what relative abundance. Advances in NGS technology now mean that the latest amplicon-based NGS protocols enable extensive multiplexing of samples, allowing researchers to process hundreds of samples and analyze millions of PCR amplicons in a single NGS-run [21].

By far the most widely used taxonomically informative gene used in such NGS methods is the 16S rRNA gene, an established genetic marker used for prokaryotic identification and classification ever since Woese and Fox first utilized rRNA sequence characterization to define the three domains of life in 1977 [22]. Sequencing of the 16S rRNA gene allows researchers to infer microbial phylogenetic relationships as the 16S rRNA gene encodes for the RNA component of the small subunit (SSU) of prokaryotic ribosomes (which perform essential functions within the translation process and is present among all bacteria and archaea) and possesses a slow rate of evolution. The 16S rRNA gene itself is approximately 1500 base pairs (bp) in size and its genetic structure comprises 9 highly conserved and 9 hypervariable regions (V1–V9). The conserved regions can serve as universal primer binding sites for the PCR amplification of gene fragments, whereas the hypervariable regions contain considerable sequence diversity, useful for prokaryotic identification purposes [23]. By comparing these hypervariable regions to 16S rRNA gene sequences of designated prokaryotic type strains available on large public databases, such as SILVA [24], RDP [25], GreenGenes [26], or NCBI [27], researchers are able to generate accurate identification of the prokaryotic taxa present within clinical samples. However, it is important to note that the accurate taxonomic identification of 16S rRNA gene data depends on the quality and completeness of the reference databases used. Most reference databases contain a number of unidentified and/or poorly annotated sequences and all reference databases are inevitably incomplete. This often frustrates an accurate taxonomic classification of 16S rRNA gene sequences [28]. Moreover, the sequencing of partial 16S rRNA genes, which is currently the most commonly used microbiota profiling strategy, often lacks the discriminatory power to differentiate prokaryotes at the species taxonomic level and is generally restricted to genus-level classification [29]. For this reason, there has been a continuous search for alternative marker genes that can improve phylogenetic resolution among prokaryotic species. For example, sequence-based analysis of the *rpoB* gene has previously been demonstrated to improve the discriminative power for characterizing prokaryotic species (when compared to 16S rRNA gene sequencing methods) among several bacterial families and genera, including *Bacillus* [30], *Enterobacteriaceae* [31], *Staphylococcus* [32], and others [33]. The *rpoB* gene encodes the highly conserved beta subunit of the prokaryotic RNA polymerase and apparently possesses the same key attributes as the 16S rRNA gene [34]. However, 16S rRNA gene sequencing studies profit from the massive amounts of sequence information already available in large publicly accessible reference databases. Hence, although alternative phylogenetic markers such as *rpoB* (and many others) are very promising [35], these biomarkers still face the challenge of competing with thousands of publications that utilize extensive databases of 16S rRNA gene sequencing information.

The characterization of eukaryotic communities is also an active research area that often employs targeted amplicon sequencing approaches. For this, the 18S rRNA gene, which is the eukaryotic nuclear homolog of the 16S rRNA gene in prokaryotes, can be used as a genetic marker to investigate fungi and protists. In fact, novel phylogenetic groups of fungal microorganisms have been defined using 18S rRNA gene-based sequencing [36], and a diversity of small eukaryotes were for the first time reported at great ocean depths (250–3000 m) using the same method [37]. Despite these efforts, in 2012, a multi-laboratory consortium proposed the nuclear ribosomal internal transcribed spacer (ITS) region as the primary genetic marker for fungi. The ITS region was preferred over the 18S rRNA gene due to the higher sequence variability found in the ITS region and the presence of a more curated and comprehensive reference database [38]. Nevertheless, it is arguable that the uneven lengths of ITS fragments may promote preferential PCR amplification of shorter ITS sequences that could lead to a biased quantification of relative abundances of fungal taxa and, therefore, the (additional) use of non-ITS targets in sequencing-based microbiota studies for fungi is desirable [39].

Finally, the detection and characterization of viruses requires a different detection approach altogether. Unlike for cellular life forms, there is not a single gene or genomic region that is homologous across all viral genomes [40]. For virus detection, microarrays that span the ‘middle ground’ between NGS and PCR-based methodologies have been developed. These microarrays are designed to detect known viruses (including phages), sometimes in combination with the simultaneous detection of prokaryotes and microbial eukaryotes [41–43]. The main advantage of these methods is the ability to simultaneously test for the presence of hundreds of viruses in a single assay and thereby remove the need for an a priori knowledge of the presence of a suspected virus. However, the range of detectable viruses is limited by the content of the viral probes that are initially spotted on the detection microarray, which may not represent the full genetic diversity of a viral community derived from a microbial sample [44].

### Shotgun metagenomics

Shotgun metagenomics is an alternative approach to characterizing microbial communities that, in contrast to targeted amplicon methods, sequences the DNA content of a clinical sample directly and produces relative abundance information for all genes detected (including for example 16S rRNA genes). This method may not only identify microorganisms per se, but may also provide information on the types of (microbial) genes present within a clinical sample, ultimately inferring functional characterization of the clinical sample. In this methodology, nucleic acids are again extracted from the sample, but are sheared into small fragments that are independently sequenced. The first shotgun metagenomics approaches to characterize microbial communities used cloned libraries to facilitate DNA sequencing via automated Sanger sequencing instruments [45, 46]. However, advances in NGS technologies mean that the cloning step is no longer necessary and greater yields of sequencing data can be obtained without this cloning bias-sensitive, labor-intensive, and costly step.

Since shotgun metagenomics is PCR-independent, and therefore not biased by primers designed to target gene sequences that are expected to be conserved within prokaryotes or small eukaryotes, the method is able to detect microorganisms that may not be detected using targeted amplicon-based NGS methods. For example, Brown and colleagues described a notable subset of bacterial taxa—known as candidate phyla radiation (CPR) bacteria—that could evade detection by 16S rRNA gene NGS methods due to self-splicing introns and proteins encoded within their rRNA genes [47]. However, four members of the *Thiotrichaceae* family are the only other bacteria outside the CPR known to have self-splicing introns within their 16S rRNA genes, illustrating their rarity in bacteria [48]. The fact that there are no broad-range genetic markers for efficient targeted amplicon sequencing of viruses means that shotgun metagenomics has revolutionized the field of virus detection and virus discovery in both clinical and environmental samples [49, 50]. Of course, the genomes of DNA viruses can be recovered through shotgun metagenomics of DNA that was directly extracted from a sample, whereas extracted RNA has to be converted to complementary DNA (cDNA) in order to detect RNA viruses [51]. Further, the low relative abundance of viral genetic material—compared to the genetic material from bacteria and host—means that it may be preferable to include a viral enrichment method prior to sequencing in order to enhance the probability of virus detection [52].

Obtaining genome sequences using shotgun metagenomics improves the researchers’ ability to discriminate microorganisms on a species-level, or even strain-level. This is in contrast to 16S rRNA gene NGS methods that offer often limited resolution at lower taxonomic levels (i.e., species and strains) due to the high sequence conservation at these taxonomic levels of the amplicons produced [29]. The identification of microbial strains is of particular importance during epidemic outbreaks caused by microorganisms, where rapid and accurate pathogen identification and characterization is essential for the management of individual cases or of entire outbreaks. For example, the genome sequence of the outbreak strain of Shiga-toxigenic *Escherichia coli* (STEC) O104:H4, which caused over 50 deaths in Germany in 2011, was reconstructed early in the outbreak using a culture-dependent whole-genome sequencing method [53]. As a result, rapid PCR screening tests were quickly developed using the available genome sequence [54, 55], which aided in tracing the source of the outbreak back to fenugreek seeds from Egypt [56]. Importantly, 2 years later, researchers were able to reconstruct the genome sequence of this outbreak strain using shotgun metagenomics directly on fecal samples that were collected from subjects during the outbreak [57]. This result highlights the potential of shotgun metagenomics to identify and characterize pathogens directly from clinical samples and supports the method’s potential diagnostic and clinical use during outbreaks of life-threatening infections caused by unknown pathogens. However, it should also be noted that the process used to reconstruct the genomes of microorganisms from such mixtures of small DNA fragments (derived from multiple microorganisms) is still very complex and requires additional bioinformatics development to further optimize sequencing resolution. This is particularly relevant for uncovering and characterizing microbial communities at the strain-level, where assembly algorithms not only have to overcome difficulties with regard to (inter-genomic) repetitive elements, but also are required to accurately incorporate small genetic differences (i.e., strain variants) that may be difficult to distinguish from actual sequencing errors [58].

Finally, shotgun metagenomics allows functional annotation of the gene sequences found within clinical samples and therefore gives a much broader description of microbial community genetics than targeted amplicon sequencing surveys. In general, functional annotation involves two steps, namely gene prediction and gene annotation. During the gene prediction step, various bioinformatics algorithms are used to determine which sequences may (partially) encode proteins. Then, once identified, these protein coding sequences are compared to a database of protein families and functionally annotated with the matching family’s function [59]. This information can then be used to discover new genes/gene sequences associated with a particular function, and/or to formulate functional pathways [60]. In this respect, it should be noted that shotgun metagenomics directed at sequencing genomic DNA does not indicate whether the predicted genes are actually being expressed within the clinical sample tested. The measurement of gene expression (via RNA sequencing) can be achieved by using metatranscriptomics approaches [61], which are beyond the scope of this review.

## Experimental pitfalls and biases

Regardless of the types of microorganisms targeted, the choices made in every step of the sequencing method used—from sample handling to data analysis—can have a serious impact on biasing the final results obtained. The effects of bias can lead to the discovery of non-existent bacterial genera, spurious correlations between microorganisms and their host, and to the lack of detection of true correlations. In this respect, there are many experimental pitfalls and biases associated with the implementation, standardization and analysis of NGS data generated using both targeted amplicon sequencing and shotgun metagenomics. Therefore, it is recommended that microbiologists use synthetic microbial community (SMC) mixes (also known as mock samples), containing multiple fully characterized microbial species, in order to calibrate their chosen protocols and identify biases introduced by their techniques [62]. In the following section, the authors focus primarily on pitfalls and biases associated with 16S rRNA gene NGS methods (although some of the pitfalls and biases also apply to shotgun metagenomics). This is because this particular method is more rapid, less complicated and cheaper to implement compared to shotgun metagenomics, and is therefore more likely to be successfully implemented into routine clinical microbiological diagnostic laboratories within a shorter timeframe. These pitfalls and biases include potential problems associated with the following 16S rRNA gene NGS steps (see also Table 1).

**Table 1:** Experimental pitfalls and biases generated using 16S rRNA gene NGS methods and their potential solutions. The potential pitfalls and biases are listed for each step of the 16S rRNA gene NGS process, from sample collection to bioinformatics analysis

Experimental pitfalls and biases	General remarks and potential solutions
Step 1: sample collection	
Transport and storage conditions can impact DNA yield and DNA quality prior to 16S rRNA gene NGS experiments.	Optimal preservation of microbial samples involves immediate freezing at − 20 °C or lower, followed by long-term storage at − 80 °C. Repeated freezing and thawing should be avoided.
Step 2: DNA extraction	
Different lysis methods can impact the final 16S rRNA gene NGS results.	The most efficient lysis method depends on the sample type and the target microbial species under investigation, which should ideally be determined by the end user. For reproducibility, the same method should be used in all subsequent experiments for this sample type.
Step 3: PCR amplification	
No 16S rRNA gene PCR primer pair is truly ‘universal’ and different primer pairs may hybridize to different proportions of ‘conserved’ sequences. The use of multi-template 16S rRNA gene PCRs inevitably generates PCR artifacts, resulting in inaccurate 16S rRNA gene NGS results.	The most optimal PCR primer pair should be selected based on its primer binding capacity to the (expected or most clinically relevant) microbial species present within the investigated sample. The use of a clonal-based amplification methodology helps limit the PCR-competition induced biases and the formation of chimeric amplicons.
Step 4: next-generation sequencing	
Current most widely used NGS-platforms produce sequence reads that span only a few hundred nucleotides, which complicates the reliable assignment of short 16S rRNA gene sequences to in silico stored reference 16S rRNA gene sequences.	Targeting the 16S rRNA gene V4 region allows for a large overlap of DNA sequences that are obtained from both ends of the PCR amplicon using Illumina’s MiniSeq/MiSeq NGS-platforms. This results in accurate NGS results with negligible error rates, though the accompanying cost is a reduction of discriminatory power due to the short amplicon size.
Step 5: Bioinformatics analysis	
The evaluation of NGS data by different bioinformatics algorithms (and their settings) may lead to different 16S rRNA gene NGS results. An accurate taxonomic identification depends on the quality and completeness of the reference databases used.	Several standardized bioinformatics pipelines are available that allow for automated sequence interpretation without the requirement for advanced bioinformatics skills. Manual evaluation of the main 16S rRNA gene NGS results is to be encouraged to ensure correct taxonomic identifications.
Miscellaneous	
16S rRNA gene NGS results are generally presented as proportional abundances of OTUs, which complicates cross-study comparability. The analysis of 16S rRNA genes is prone to the introduction of contaminating DNA derived from the experimental set-up during sample processing.	The use of protocols that determine the absolute quantity of OTUs improves the standardization of 16S rRNA gene NGS results in different studies. An adequate number of negative (extraction) control samples should be included and analyzed to identify (and remove) any 16S rRNA gene copies originating from contaminating DNA.

*OTU* operational taxonomic units—a group of very similar sequences

### Sample handling

The choice of the most optimal sampling protocol depends on the sample type to be investigated. However, all samples have to be transported to the relevant routine clinical microbiological diagnostic laboratory and stored for a certain period of time before these samples are processed. The transport and storage conditions of clinical samples are important factors that can have an impact on DNA yield and DNA quality prior to targeted amplicon sequencing investigations. In this respect, several studies have evaluated how different storage and transit conditions may affect the stability of the microbial composition. For example, Carroll et al. demonstrated the microbial stability of fecal samples over a 24-h period at room temperature and 6 months of long-term storage at − 80 °C [63]. Others have shown that storage of fecal samples for 3 days at room temperature did not affect total DNA purity and relative 16S rRNA gene contents [64], but that DNA became fragmented when samples were inconsistently freeze thawed or when samples had been kept for over 2 weeks at room temperature [65]. Interestingly, although a recent study by Shaw et al. indicated that fecal samples stored for more than 2 years at − 80 °C are still largely representative of the original microbial community composition [66], there is compelling quantitative PCR (qPCR) evidence to indicate that *Bacteroides* species present within fecal samples are already reduced after 1 week of storage at − 20 °C [67, 68]. Moreover, samples other than feces, including sputum samples, also showed significant distortions in their microbiota profiles; (1) after short storage at room temperature and (2) after being freeze thawed several times [69, 70]. Therefore, the most optimal preservation of microorganisms for accurate microbiota profiling during sampling, transport, and storage appears to involve immediate freezing at ≤ − 20 °C, followed by long-term storage at − 80 °C [71].

### DNA extraction

All DNA-based methods, including 16S rRNA gene NGS methods, rely on the effective lysis of microorganisms to liberate genomic material for downstream analysis. In order to achieve effective lysis, several procedures have been developed, including chemical lysis, ‘bead-beating’ (the mechanical disruption of cells), lysis using detergents, or a combination of these approaches. However, some cell types may resist common mechanical or chemical lysis methods that may result in important differences in the performance of commercially available DNA extraction kits [72, 73]. For example, some methods have been previously shown to yield a reduced recovery of Gram-positive microorganisms compared to Gram-negative microorganisms (presumably due to differences in the composition of the respective microbial cell envelopes) [74], and an effective cell lysis becomes even more problematic for microorganisms whose cell envelope contains the difficult to lyse component mycolic acid, such as mycobacteria [75]. Essentially, the choice of the most optimal DNA extraction method is greatly dependent on the sample type and target microbial species to be investigated, but in any case, should ideally be determined within each individual laboratory for its own purpose. Once determined, the same protocol should be employed consistently for similar clinical samples.

### Contaminating DNA

The validity of targeted amplicon sequencing results is threatened by the presence of contaminating DNA derived from the (laboratory) environment and/or the reagents/consumables used during sample processing. For example, PCRs may yield billions of amplicons, which combined with the extreme sensitivity of PCR amplification, means that there is a high risk of amplicon contamination within research and routine clinical microbiological diagnostic laboratories that regularly use PCR. For this reason, many laboratories spatially separate pre- and post-PCR steps in order to limit the risk of amplicon cross-contamination between distinct PCR experiments. Additionally, Glassing et al. showed that commercially available DNA extraction and PCR amplification kits may generate up to 20,000 16S rRNA gene sequences, representing more than 80 prokaryotic genera, even without the addition of any sample [76]. These contamination issues are especially important for the accurate analysis of the microbial composition of low biomass samples, for example joint fluids, cerebrospinal fluids, blood samples, or other samples derived from normally ‘sterile’ body sites. Salter et al. clearly illustrated how contaminating DNA can affect the microbiota results obtained [77]. The researchers sequenced a pure culture of the bacterium *Salmonella bongori* as well as a series of diluted versions and showed that DNA contamination increased with each dilution and quickly drowned out the original *S*. *bongori* signal. Therefore, in order to minimize the chance of erroneous conclusions derived from sequencing clinical samples, it is essential that negative extraction controls (specifically, template-free ‘blanks’ processed with the same DNA extraction, and PCR amplification kits as the actual samples) be included in 16S rRNA gene NGS protocols in order to allow for the identification of amplicon sequences that originate from DNA contamination.

### Selection of 16S rRNA gene PCR primers

Universal 16S rRNA gene PCR primer sets are designed to specifically amplify conserved 16S rRNA gene sequences from as many prokaryotic species as possible. However, it is well-known that there are no suitable 100% conserved regions of the 16S rRNA gene available for PCR amplification. This lack of sequence conservation can lead to inaccurate microbial sequence detection due to inefficient PCR primer binding. In order to ensure the detection of the specific microbial taxa of interest in a particular study, several researchers have reported on the adaptation of universally applicable 16S rRNA gene PCR primer sets via the introduction of degenerate base pairs at the positions of 16S rRNA gene/primer sequence mismatches [78, 79]. In addition, the multiple hypervariable regions of each 16S rRNA gene exhibit different degrees of sequence diversity that varies from genus to genus, resulting in an ongoing debate about the most efficient hypervariable regions to be used for accurate phylogenetic analysis and taxonomic classification, as none is perfect [80, 81]. However, the choice for a particular hypervariable region also depends on the technological limitations of the NGS-platforms used. For example, the short length of the 16S rRNA gene V4 region (~ 250 bp) allows for a full overlap of DNA sequences that are obtained from both ends of the PCR amplicon using Illumina’s MiSeq NGS-platform, which is currently the most commonly used NGS-platform. This strategy generates the lowest error rates, which have resulted in more accurate diversity estimates, compared to the results obtained from the not completely overlapping V3–V4 and V4–V5 regions, though the accompanying cost is a reduction of discriminatory power due to the shorter amplicon size [21]. With this in mind, the amplification and sequencing of multiple hypervariable regions [62], or even the generation of (near) full-length 16S rRNA gene sequences using upcoming third-generation sequencing platforms [82, 83], give the most complete description of microbiota profiles within a microbial sample. However, the applicability of these third-generation sequencing platforms is still far from certain due to high costs per sample, low throughput, and relatively high base-calling error rates [84].

### PCR competition effects

Although often neglected in 16S rRNA gene NGS studies, PCR is a competitive process meaning that the presence of multiple 16S rRNA gene template molecules in a single reaction tube may lead to the preferential amplification of a subset of 16S rRNA gene targets that amplify more efficiently compared to other 16S rRNA gene targets [85]. These differences in template DNA amplification efficiencies may lead to inaccurate microbiota profiling results within clinical samples. There are several mechanisms (relating to the differences in 16S rRNA gene target sequence composition) that could lead to such preferential PCR amplification, including primer binding capacity, sequence length, and GC-content [85, 86]. However, compensating for these different amplification efficiencies requires optimized PCR conditions that guarantee equal amplification efficiency for each individual 16S rRNA gene target, which is practically impossible when investigating polymicrobial clinical samples of unknown composition. An extra complication based on our own experience investigating clinical samples is that PCR amplification efficiencies of 16S rRNA gene targets may be reduced in samples that contain high levels of human DNA and low levels of prokaryotic DNA, probably via the formation of competing non-specific amplicons [87]. Thus, although NGS is a very sensitive detection platform, differences in PCR amplification efficiency of 16S rRNA gene targets within a polymicrobial clinical sample may lead to a biased (and even false) outcome of the original clinical sample composition. Therefore, methodological steps should be taken to reduce the effect of PCR amplification efficiency bias.

### Chimera formation

16S rRNA gene PCRs will generate chimeric amplification products (whereby a single DNA amplicon comprises sequences that originate from multiple different 16S rRNA genes), which may be falsely interpreted as a novel microorganism or an existing but absent microorganism, thus inflating the apparent sample richness (i.e., the number of microbial taxa present within a sample). The most commonly described mechanism of chimera formation involves prematurely terminated PCR products that can serve as PCR primers to amplify related template DNA molecules on subsequent PCR cycles [88]. In addition, chimera formation might also occur due to template-switching events during DNA synthesis [89], or via the incorporation of random DNA fragments, such as shortened PCR primers and degraded amplicons that might be produced by proofreading enzymes during PCR amplification [90]. Importantly, chimeras are frequent artifacts in 16S rRNA gene NGS studies and have been detected at a frequency of up to 30%, although the frequency of chimera production decreases, as expected, when template DNA similarity diminishes [91]. In order to reduce the chance of chimera formation, optimized PCR protocols have been proposed that include the use of a highly processive polymerase and a minimized number of PCR cycles [92], but no method has been shown to eliminate these artifacts entirely. In addition, numerous computational approaches have been developed over the years to detect and remove chimeric sequences from 16S rRNA gene NGS datasets [88, 93–95], but these different methods often disagree with one another [88, 96]. Thus, chimeras continue to be a major cause of concern to researchers performing 16S rRNA gene NGS research. Even more disturbing, public 16S rRNA gene reference databases are already suspected of containing a significant number of chimeric sequences that further complicate the reliable taxonomic classifications obtained from 16S rRNA gene NGS research and diagnosis [94]. Optimized methodologies need to be developed that reduce the generation of chimeric amplification products without relying on bioinformatics-based chimera identification and filtering steps.

### Bioinformatics analysis

The analysis of 16S rRNA gene NGS data requires an extensive array of bioinformatics algorithms that are involved in computational intensive steps such as quality filtering, operational taxonomic unit (OTU) clustering, and sequence classification. Currently, there are many different bioinformatics algorithms available for this purpose, which makes it difficult for non-bioinformatics educated users—including most technicians in routine clinical microbiological diagnostic laboratories—to identify the most accurate approaches for 16S rRNA gene NGS analysis. Importantly however, multiple studies have shown that the choice of certain bioinformatics algorithms and their settings can affect the final microbiota results obtained [97, 98]. For this reason, popular open-source programs, such as mothur and QIIME, have aided in these issues via the re-writing of specific bioinformatics algorithms (e.g., mothur) or combining original published bioinformatics algorithms (e.g., QIIME) into single optimized software packages [99, 100]. These programs have excellent online tutorials and forums to further support the (inexperienced) user, but their use remains complex as both programs have implemented a collection of command-line tools that represent a large number of bioinformatics algorithms and settings. Interestingly, several automatic, ‘easy-to-use’ bioinformatics pipelines have been developed recently that are (partially) built on the bioinformatics algorithms available in mothur and/or QIIME and enable the analysis of 16S rRNA gene NGS data without knowledge of command-line scripts that would normally be required [101, 102]. However, none of these have been specifically designed for use in the routine clinical diagnostic microbiological laboratory and represent only a part of the NGS processing and analysis pipeline required to generate accurate results.

Recently, there is a discussion about whether the OTU-based approaches used by mothur (average linkage) and QIIME (uclust) should be replaced by newly developed amplicon sequence variants (ASV)-based methods as the standard to delineate microbial taxa [103]. These ASV-based methods avoid clustering sequences at the arbitrary thresholds that are currently used to define OTUs (e.g., 97%) by using only unique, identical sequence reads for downstream analysis. Unlike OTUs, ASVs can be resolved down to the level of single-nucleotide differences over the sequenced gene region that is expected to increase taxonomic resolution [104], although it could be argued that the fine-scale resolution obtained using ASV-based methods is actually not always desirable when processing highly complex samples. For example, the increased resolution of ASVs may increase the alpha diversity and reduce the overlap between samples, making downstream analyses more difficult compared to sequences that have been clustered into OTUs [105]. Further, OTU- and ASV-based methods will often produce comparable ecological results when the 16S rRNA gene is used as a genetic marker [106]. This finding can be explained by the fact that 16S rRNA gene sequence types may not reflect ecologically or phylogenetically cohesive populations [107].

## MYcrobiota—an ‘end-to-end’ 16S rRNA gene NGS platform for routine clinical microbiological diagnostics

In order to successfully implement 16S rRNA gene NGS technology within the routine clinical microbiological diagnostic laboratory, it is essential to first understand and apply solutions to the pitfalls and biases associated with this particular technology. With this in mind, the authors developed and published a standardized 16S rRNA gene NGS platform (‘MYcrobiota’), which was designed to overcome the most important experimental pitfalls and biases of current 16S rRNA gene NGS methods that have previously hampered the introduction of these methods into the routine clinical diagnostic laboratories (Table 1) [16]. MYcrobiota is a consolidated tool that includes a novel micelle PCR/NGS (micPCR/NGS) methodology and a dedicated, easy-to-use bioinformatics pipeline that was specifically designed for use in routine clinical microbiological diagnostic laboratories. The micPCR/NGS method drastically reduces chimera formation compared with traditional 16S rRNA gene NGS methods and prevents PCR competition effects via the clonal amplification of targeted 16S rRNA gene molecules [108]. Importantly, by adding an internal calibrator to the micPCR/NGS methodology, MYcrobiota allows the researcher to express the resulting OTUs detected within the clinical sample under investigation as a measure of 16S rRNA gene copies, which also enables the subtraction of any non-sample associated contaminating 16S rRNA gene copies per OTU that have been derived from laboratory reagents and/or the laboratory environment [87]. This means that MYcrobiota possess a much higher accuracy and a lower limit of detection (LOD) compared to (semi-quantitative) traditional 16S rRNA gene NGS methods, allowing the accurate detection of bacterial OTUs at very low abundances (for example in low biomass clinical samples), or alternatively, can reliably confirm the absence of bacterial DNA in culture-negative clinical samples. Essentially, if the number of specific 16S rRNA gene copies in a clinical sample is greater than the number of the same 16S rRNA gene copies in the negative controls, then the result is more likely to be a true positive result for the actual presence of that specific microorganism in the clinical sample. Further, the bioinformatics pipeline that is part of MYcrobiota enables the full (automatic) analyses of the micPCR/NGS data obtained from raw sequence files to final web reports that summarizes the microbiota results, together with an extensive overview of the quality control measurements performed during the data analysis [16, 109]. Of course, although MYcrobiota shows potential as a means of successfully adapting 16S rRNA gene NGS from research tool to reliable routine clinical microbiological diagnostic, the authors appreciate that factors such as costs, user training, quality assurance, connectivity with laboratory information management systems, etc. also play a role in the implementation of novel diagnostics into clinical use [110]. However, the implementation of novel diagnostic tools such as MYcrobiota (that have the ability to detect, quantify, and characterize bacterial DNA derived from live, fastidious, and dead bacterial cells present within (polymicrobial) clinical samples) is a necessary step in allowing such general discussions to begin.

## Conclusions

Culture-independent NGS methods, such as targeted amplicon sequencing (e.g., 16S rRNA gene NGS) and shotgun metagenomics, have the potential to greatly impact on routine clinical microbiological diagnostic laboratories by detecting DNA derived from live, fastidious, and dead bacterial cells present within clinical samples. Such results could potentially be used to benefit patients by influencing antibiotic prescribing practices [111], or to generate new classical-based diagnostic tests (e.g., novel culture or PCR diagnostics). However, experimental pitfalls and biases in current NGS protocols, together with the requirement for access to bioinformaticians, currently hinders the introduction of NGS methods into routine clinical microbiological diagnostics. This review article outlines the main pitfalls and biases to the successful implementation of 16S rRNA gene NGS and provides several relevant solutions for microbiologists to consider. Finally, we introduce a published 16S rRNA gene NGS platform that incorporates these solutions, in order to overcome such potential pitfalls and biases as PCR competition effects, chimera formation and DNA contamination, while at the same time providing an easy-to-use bioinformatics pipeline that allows for automated 16S rRNA gene NGS interpretation without the need for advanced bioinformatics skills. Although the development of MYcrobiota is only one of many steps required for the successful implementation of novel diagnostics into the routine clinical microbiological diagnostic laboratory, the knowledge and understanding of the principles outlined in this publication will help to fill the gap between traditional ‘gold standard’ microbiological methods (culture and PCR) and the as yet unfulfilled potential of NGS technologies in routine clinical microbiological diagnostics.
